# Associations of Life‐Course Social Isolation Trajectories and Depressive Symptoms With the Risk of Incident Cardiovascular Disease: A Prospective Cohort Study

**DOI:** 10.1155/da/2184277

**Published:** 2026-04-08

**Authors:** Shuaiqing Chen, Peiling Jiang, Qiuxia Zheng

**Affiliations:** ^1^ Department of Cardiology, Lishui Hospital of Traditional Chinese Medicine, Lishui, China, guahao.zjol.com.cn; ^2^ Department of Critical Care Medicine, Lishui Hospital of Traditional Chinese Medicine, Lishui, China, guahao.zjol.com.cn; ^3^ Department of Nursing, Lishui Hospital of Traditional Chinese Medicine, Lishui, China, guahao.zjol.com.cn

**Keywords:** cardiovascular disease, depressive symptoms, life-course, social isolation, stroke

## Abstract

**Background:**

Social isolation is increasingly recognized as a fundamental determinant of health. This study aimed to comprehensively examine the associations between social isolation trajectories spanning from childhood through adulthood and the risk of incident cardiovascular disease (CVD) among middle‐aged and older adults in China, while further exploring the potential mediating role of depressive symptoms in these relationships.

**Methods:**

We analyzed data from 6858 participants using the 2014 life‐course survey and 2015–2020 follow‐up waves of the China Health and Retirement Longitudinal Study (CHARLS). Trajectories were constructed based on the cross‐classification of social isolation status in childhood and adulthood. Exposures were assessed using cumulative composite scores, and incident CVD outcomes were identified via self‐reported physician diagnoses. Multivariable logistic regression models were applied to estimate the odds of incident CVD, alongside mediation analyses to quantify potential indirect effects. Subgroup analyses and a comprehensive set of sensitivity analyses were additionally conducted to assess the robustness of the findings.

**Results:**

Four distinct life‐course social isolation trajectories were identified: no isolation, childhood‐only, adulthood‐only, and persistent isolation. Persistent social isolation was associated with significantly elevated odds of incident CVD (OR = 1.53, 95% CI: 1.16–2.01). Mediation analyses indicated that depressive symptoms accounted for a meaningful proportion of this association, explaining 16.28% of the relationship with CVD and 14.70% of the association with stroke. Analyses of secondary outcomes further demonstrated that childhood social isolation (OR = 1.44, 95% CI: 1.16–1.79), childhood‐only isolation (OR = 1.36, 95% CI: 1.08–1.72), and persistent isolation (OR = 1.79, 95% CI: 1.19–2.71) were each independently associated with increased odds of incident stroke.

**Conclusions:**

Persistent social isolation was associated with an increased risk of incident CVD among middle‐aged and older adults in China, with this relationship being partially mediated by depressive symptoms. These findings underscore the importance of incorporating long‐term psychosocial assessments into CVD risk stratification and management strategies.

## 1. Introduction

Cardiovascular disease (CVD) remains the foremost contributor to mortality at the global level [1, 2]. Recent estimates from the Global Burden of Disease study indicate a sustained increase in CVD‐related deaths from 1990 to 2022, underscoring a growing global public health challenge [3]. In China, this burden is particularly pronounced amid rapid population aging and profound lifestyle transitions; according to the China Cardiovascular Health and Disease Report 2022, CVD has become the foremost cause of death in both urban and rural populations [4]. Although prevention and management of traditional clinical risk factors, such as hypertension and dyslipidemia, have achieved measurable progress, contemporary clinical strategies continue to prioritize biomedical interventions. The limitations of this narrow focus are increasingly evident, as it overlooks the broader social contexts in which health is shaped. Consequently, authoritative organizations, including the American Heart Association, have recently called for a paradigm shift toward the social determinants of health as a critical avenue for advancing CVD prevention and control [5, 6].

As a core component of the social determinants of health, social isolation is defined as the objective absence of social contacts or supportive relationships [7]. The World Health Organization has reframed social isolation as an urgent health concern rather than a purely social issue [8]. In a landmark advisory released in 2023, the US Surgeon General cautioned that the mortality risk associated with social isolation is comparable to that of smoking and obesity [9]. Although a substantial body of literature has linked social isolation to an elevated risk of CVD, most prior investigations have relied on single time‐point assessments conducted in adulthood [10, 11]. Such a static approach is inherently limited in its ability to capture the dynamic evolution and cumulative impact of social isolation across the life course. From a life‐course epidemiological perspective, early‐life experiences such as social withdrawal or bullying may become biologically embedded, interacting with adverse exposures in later life to generate a cumulative disease burden that exceeds the simple aggregation of risks at isolated periods [12]. Despite this theoretical framework, empirical evidence delineating how social isolation trajectories from childhood through adulthood shape the development of CVD remains scarce.

Prior research indicates that sustained social isolation can function as a chronic stressor, provoking systemic inflammatory activation and neuroendocrine dysregulation, thereby accelerating cardiovascular pathological processes [13, 14]. Depressive symptoms, a frequent psychological sequela of social isolation, have been closely linked to chronic low‐grade inflammation and elevated CVD risk [15, 16]. However, its mediating role in the association between life‐course social isolation trajectories and incident CVD has yet to be quantitatively delineated. Moreover, it remains unclear whether persistent isolation confers a greater pathogenic burden than transient forms of isolation.

Against this backdrop, the present study leveraged high‐quality, nationally representative data from the China Health and Retirement Longitudinal Study (CHARLS), integrating unique retrospective life‐course information to address these critical knowledge gaps. We hypothesized that persistent social isolation across the life course would be strongly associated with an increased risk of incident CVD and that depressive symptoms would partially mediate this relationship. The findings are expected to provide robust epidemiological evidence to inform more precise cardiovascular risk stratification and the development of early psychosocial intervention strategies among middle‐aged and older adults in China.

## 2. Methods

### 2.1. Study Design and Participants

This analysis drew on data from the CHARLS, a nationally representative prospective cohort designed to track demographic, socioeconomic, and health trajectories among Chinese adults aged 45 years and older [17]. The study was initiated in 2011–2012, with subsequent follow‐up assessments conducted in 2013, 2015, 2018, and 2020. Notably, a comprehensive life‐course survey was implemented in 2014, and venous blood samples were collected in 2012 and 2015 for biomarker assessment. All interviews were administered by trained personnel using a computer‐assisted personal interviewing (CAPI) system. The protocol of the CHARLS was approved by the Biomedical Ethics Review Committee of Peking University (approval number: IRB00001052‐11015), with written informed consent provided by all participants at the time of study entry.

**Figure 1:**
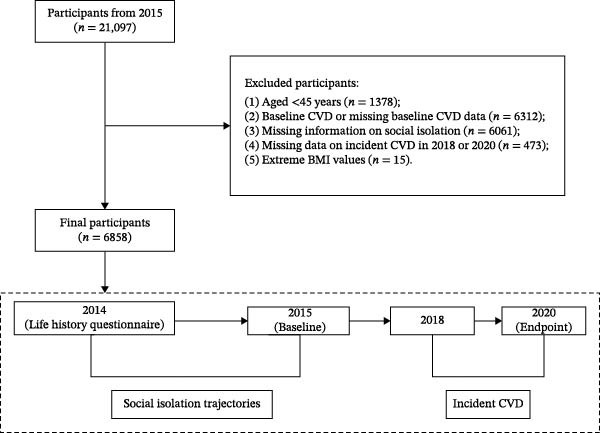
Flowchart of participant selection and schematic of the study design.

To integrate adult assessments with childhood histories collected in 2014, the 2015 wave was designated as the analytical baseline, with outcomes ascertained during follow‐up in 2018 and 2020. Of the 21,097 individuals who participated in the 2015 survey, a total of 6858 participants were ultimately included in the present analysis (Figure 1). Participants were excluded if they met any of the following criteria: age younger than 45 years; prevalent CVD at baseline or missing baseline CVD information; incomplete data on childhood or adulthood social isolation; missing information on incident CVD outcomes in 2018 or 2020; or extreme body mass index (BMI) values. Baseline characteristics of included and excluded participants are presented in Table S1.

### 2.2. Exposure Assessment

We characterized social isolation across the life course by creating a composite trajectory variable that integrated information from both childhood and adulthood. Childhood isolation was derived from the 2014 life‐course survey, which operationalized Caspi’s framework of social withdrawal and exclusion [18]. Childhood was defined as the period before age 17, consistent with the CHARLS Life History Survey design. Four items were assessed: having a group of friends and feeling lonely, which captured withdrawal, and being bullied by neighbors or school peers, which reflected exclusion. Each item was scored on a 3‐point scale. The item on having friends was reverse coded (often = 0; sometimes = 1; rarely or never = 2), whereas the remaining items were scored in the forward direction (rarely or never = 0; sometimes = 1; often = 2). Scores were summed to yield a cumulative index ranging from 0 to 8, and values of 2 or higher were classified as childhood social isolation. This cut‐off value was determined based on the distribution of the cumulative scores and is consistent with previous validated studies utilizing CHARLS data [19]. Adulthood isolation was assessed at the 2015 baseline using a composite index comprising four binary indicators: being unmarried, living alone, absence of weekly contact with children, and lack of social participation [20]. Adulthood refers to the participant’s age at the 2015 baseline. A cumulative score of 2 or higher (range 0–4) indicated adulthood social isolation. Consistent with prior research [21], we derived four mutually exclusive trajectories of social isolation using a concept‐driven cross‐classification approach, combining isolation status across the two life stages. These trajectories were defined as no isolation, childhood‐only isolation, adulthood‐only isolation, and persistent isolation.

### 2.3. Mediating Variables

We postulated that depressive symptoms represent a key pathway linking social isolation trajectories to the onset of CVD and therefore evaluated them in detail as a potential mediator. Depressive symptoms were assessed at the 2015 baseline using the 10‐item short form of the Center for Epidemiologic Studies Depression Scale (CES‐D‐10), an instrument with well‐established reliability and validity that has been extensively applied to evaluate mental health among middle‐aged and older adults in China [22, 23]. The scale comprises 10 items capturing the frequency of specific affective and behavioral experiences during the preceding week, encompassing domains such as depressive mood, fearfulness, loneliness, sleep disturbance, and difficulty concentrating. Each item is rated on a 4‐point scale, ranging from “rarely or none of the time (<1 day)” scored as 0, to “most or all of the time (5–7 days)” scored as 3. Two positively worded items (“I felt hopeful” and “I felt happy”) are reverse‐coded to ensure accurate scoring. Scores range between 0 and 30, with increasing values reflecting a higher burden of depressive symptoms. Consistent with prior validation studies using CHARLS data and established clinical thresholds, a total score of 10 or higher was used to define the presence of clinically meaningful depressive symptoms [24].

### 2.4. Outcome Definition

The primary outcome was defined as incident CVD. Outcome status was ascertained on the basis of participant self‐reports of physician‐diagnosed disease during the 2018 and 2020 follow‐up waves. Incident CVD was ascertained among participants who reported a new diagnosis of heart disease or stroke during follow‐up. To ensure the incident nature of the outcome, individuals who self‐reported a history of CVD at baseline in 2015 were rigorously excluded. In addition, incident heart disease and incident stroke were analyzed separately as secondary outcomes.

### 2.5. Covariates

To control for potential confounding, a comprehensive set of baseline covariates was selected on the basis of prior literature and clinical relevance. Demographic characteristics included age, sex, education (categorized as below elementary, elementary, and above elementary), and residence (urban or rural). Lifestyle factors and anthropometric indicators comprised smoking, drinking, and BMI. Baseline health conditions included the presence of hypertension, diabetes, and dyslipidemia. Early‐life context was characterized using childhood household economic status and self‐rated childhood health, each classified as good, fair, or poor. Patterns of missing data across all covariates are summarized in Figure S1.

### 2.6. Statistical Analysis

Baseline characteristics were described after stratification by social isolation trajectories. Continuous data were described using means and standard deviations (SDs) and analyzed with analysis of variance (ANOVA), whereas categorical data were presented as counts and percentages, with group comparisons performed using Pearson chi‐square tests or Fisher exact tests as appropriate. To minimize potential bias and maximize data utilization, missing values in covariates were addressed using multiple imputation by chained equations (MICE), generating five complete datasets; estimates were subsequently pooled according to Rubin’s rules.

Variance inflation factors (VIFs) were estimated before model specification to examine possible multicollinearity across covariates. Multivariable logistic regression models were then fitted to estimate odds ratios (ORs) and 95% confidence intervals (CIs) for the associations of childhood isolation, adulthood isolation, and life‐course social isolation trajectories with incident CVD, using the no‐isolation group as the reference. A hierarchical modeling strategy was applied: Model 1 was unadjusted. Model 2 adjusted for all baseline confounders (demographic characteristics, early‐life factors, lifestyle variables, and baseline health conditions) to estimate the total effect of social isolation on incident CVD. Model 3 further incorporated depressive symptoms to observe the attenuation of the effect size, estimating the direct effect and serving as a conceptual basis for the subsequent formal mediation analysis. Subsequently, exploratory mediation analyses were conducted to quantify the mediating role of depressive symptoms, with adjustment for all covariates included in Model 2. Total effects, natural direct effects, and natural indirect effects were estimated, and the proportion mediated by depressive symptoms was calculated.

To further assess the robustness of the primary findings, a series of subgroup and sensitivity analyses were conducted. Subgroup analyses stratified the fully adjusted cohort by sex, residence, and depressive symptoms, with interaction tests performed to evaluate potential effect modification. A series of rigorous sensitivity analyses were implemented: first, to fully leverage follow‐up time information, multivariable Cox proportional hazards models were constructed as a supplementary analysis to estimate hazard ratios (HRs) and 95% CI, thereby assessing the temporal consistency of the main results. Second, to evaluate potential bias introduced by multiple imputation, complete‐case analyses were performed, in which regression models were re‐run using only participants with complete covariate data. Finally, recognizing that residual confounding cannot be entirely excluded in observational studies, E‐values were calculated to quantify the potential impact of unmeasured confounders on the observed associations.

All analyses were performed using R software (version 4.2.2), and two‐sided *p* < 0.05 was considered statistically significant.

## 3. Results

### 3.1. Basic Characteristics of Participants

The final analytic cohort comprised 6858 participants with a mean age of 59.20 ± 8.46 years, of whom 55.42% were men. The distribution of life‐course social isolation trajectories was as follows: no isolation (*n* = 4237, 61.8%), childhood‐only isolation (*n* = 1795, 26.2%), adulthood‐only isolation (*n* = 503, 7.3%), and persistent isolation (*n* = 323, 4.7%). The dynamic transitions and proportional flow of participants across these distinct social isolation statuses from childhood to adulthood are visually detailed in Figure S2. As shown in Table 1, participants in the persistent isolation group were older, had lower educational attainment, and were more likely to reside in rural areas compared with those in the no‐isolation group. With respect to early‐life context, persistent isolation was characterized by the highest proportions of disadvantaged childhood household economic status (48.30%) and poor self‐rated childhood health (14.55%). Notably, although the burden of chronic metabolic conditions was broadly comparable across trajectory groups, marked differences were observed in mental health status. Specifically, the prevalence of depressive symptoms in the persistent isolation group reached 43.96%, substantially exceeding that observed in the no‐isolation group (24.64%).

**Table 1:** Baseline characteristics of participants stratified by social isolation trajectories.

Variables	Total(*n* = 6858)	No isolation(*n* = 4237)	Childhood only (*n* = 1795)	Adulthood only (*n* = 503)	Persistent isolation (*n* = 323)	*p*‐Value
Age, years	59.20 ± 8.46	58.28 ± 8.09	59.97 ± 8.57	61.63 ± 9.23	63.05 ± 9.17	<0.001
BMI (kg/m)^2^	23.85 ± 3.83	24.07 ± 3.77	23.51 ± 3.90	23.61 ± 3.97	23.22 ± 3.80	<0.001
Sex *n* (%)						0.003
Female	3057 (44.58)	1910 (45.08)	746 (41.56)	253 (50.30)	148 (45.82)	
Male	3801 (55.42)	2327 (54.92)	1049 (58.44)	250 (49.70)	175 (54.18)	
Education *n* (%)						<0.001
Below elementary	2039 (29.73)	998 (23.55)	718 (40.00)	166 (33.00)	157 (48.61)	
Elementary	1996 (29.10)	1227 (28.96)	524 (29.19)	145 (28.83)	100 (30.96)	
Above elementary	2823 (41.16)	2012 (47.49)	553 (30.81)	192 (38.17)	66 (20.43)	
Residence, *n* (%)						<0.001
Urban	1325 (19.32)	937 (22.11)	268 (14.93)	80 (15.90)	40 (12.38)	
Rural	5533 (80.68)	3300 (77.89)	1527 (85.07)	423 (84.10)	283 (87.62)	
Smoking, *n* (%)						0.958
No	4629 (67.50)	2852 (67.31)	1216 (67.74)	344 (68.39)	217 (67.18)	
Yes	2229 (32.50)	1385 (32.69)	579 (32.26)	159 (31.61)	106 (32.82)	
Drinking, *n* (%)						<0.001
No	4101 (59.80)	2470 (58.30)	1103 (61.45)	337 (67.00)	191 (59.13)	
Yes	2757 (40.20)	1767 (41.70)	692 (38.55)	166 (33.00)	132 (40.87)	
Hypertension, *n* (%)						0.956
No	5047 (73.59)	3128 (73.83)	1313 (73.15)	369 (73.36)	237 (73.37)	
Yes	1811 (26.41)	1109 (26.17)	482 (26.85)	134 (26.64)	86 (26.63)	
Diabetes, *n* (%)						0.710
No	6326 (92.24)	3900 (92.05)	1665 (92.76)	466 (92.64)	295 (91.33)	
Yes	532 (7.76)	337 (7.95)	130 (7.24)	37 (7.36)	28 (8.67)	
Dyslipidemia, *n* (%)						0.449
No	5824 (84.92)	3575 (84.38)	1541 (85.85)	430 (85.49)	278 (86.07)	
Yes	1034 (15.08)	662 (15.62)	254 (14.15)	73 (14.51)	45 (13.93)	
Depression symptoms, *n* (%)						<0.001
No	4846 (70.66)	3193 (75.36)	1138 (63.40)	334 (66.40)	181 (56.04)	
Yes	2012 (29.34)	1044 (24.64)	657 (36.60)	169 (33.60)	142 (43.96)	
Childhood economic status, *n* (%)						<0.001
Good	625 (9.11)	435 (10.27)	119 (6.63)	52 (10.34)	19 (5.88)	
Fair	3633 (52.97)	2403 (56.71)	802 (44.68)	280 (55.67)	148 (45.82)	
Poor	2600 (37.91)	1399 (33.02)	874 (48.69)	171 (34.00)	156 (48.30)	
Childhood health status, *n* (%)						<0.001
Good	2525 (36.82)	1676 (39.56)	576 (32.09)	174 (34.59)	99 (30.65)	
Fair	3522 (51.36)	2132 (50.32)	941 (52.42)	272 (54.08)	177 (54.80)	
Poor	811 (11.83)	429 (10.13)	278 (15.49)	57 (11.33)	47 (14.55)	

*Note*: Data are presented as mean ± standard deviation (SD) or number (%), as appropriate. The *p*‐Value is based on analysis of variance (ANOVA), chi‐square test, or Fisher’s exact test.

Abbreviation: BMI, body mass index.

### 3.2. Associations of Social Isolation With Incident CVD

As illustrated in Figure 2, the incidence of CVD differed markedly across social isolation trajectories. The lowest incidence was observed in the no‐isolation group (16.8%), followed by the childhood‐only isolation group (19.0%) and the adulthood‐only isolation group (18.7%), whereas the persistent isolation group exhibited the highest incidence, reaching 24.8%. A similar gradient was evident for specific cardiovascular outcomes, with the persistent isolation group showing the highest rates of incident heart disease (17.0%) and incident stroke (9.6%).

Figure 2Proportion of incident heart disease (A), stroke (B), and CVD (C) stratified by social isolation trajectories.

**Figure:**
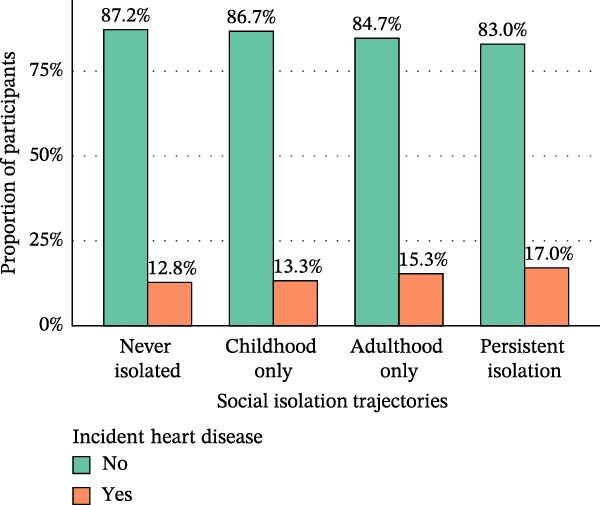
(A)

**Figure:**
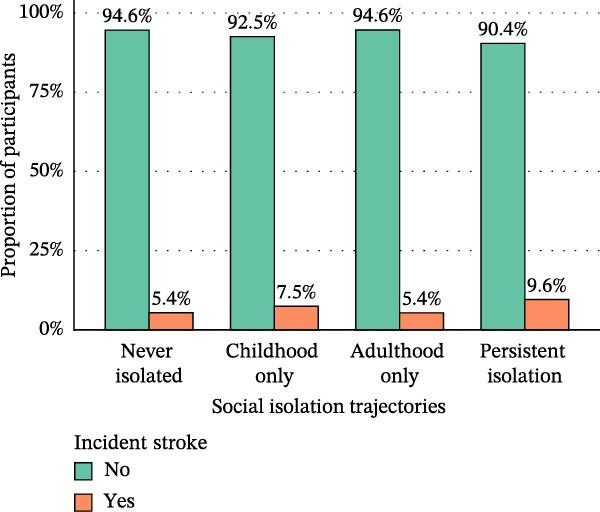
(B)

**Figure:**
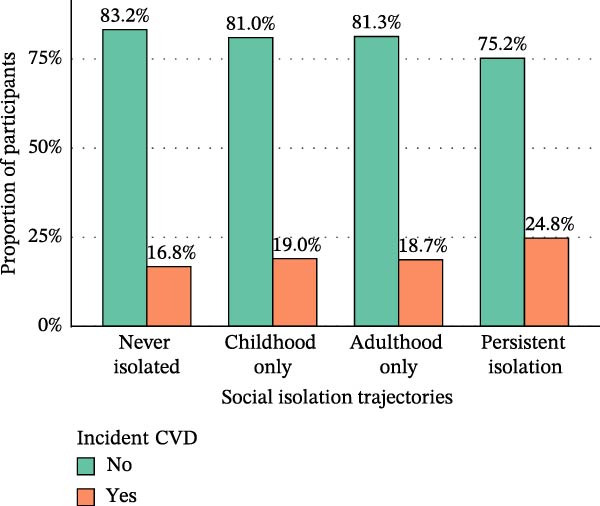
(C)

Diagnostic assessments conducted prior to model fitting indicated that all covariates had VIF below 2, suggesting no evidence of multicollinearity (Table S2). Detailed results of the logistic regression analyses are presented in Table 2. When social isolation at specific life stages was examined separately, childhood isolation was significantly associated with incident CVD (OR 1.18, 95% CI 1.03–1.36) in the fully adjusted model accounting for baseline confounders (Model 2), whereas adulthood isolation was not (OR 1.17, 95% CI 0.97–1.41). In contrast, analyses of life‐course social isolation trajectories revealed that, compared with the no‐isolation group, participants experiencing persistent isolation had the highest odds of incident CVD in the unadjusted model (Model 1: OR 1.64, 95% CI 1.25–2.13). In Model 2, persistent isolation was significantly associated with increased odds of incident CVD (OR 1.53, 95% CI 1.16–2.01), representing the total effect. Further incorporation of depressive symptoms in Model 3 attenuated the effect size (OR 1.42, 95% CI 1.08–1.88). Collectively, these findings suggest that only cumulative exposure to social isolation across the life course is associated with a meaningful increase in the odds of CVD.

**Table 2:** Association of social isolation trajectories with incident CVD.

Variables	Model 1		Model 2		Model 3	
	OR (95% CI)	*p*‐Value	OR (95% CI)	*p*‐Value	OR (95% CI)	*p*‐Value
Childhood social isolation						
No	Ref	—	Ref	—	Ref	—
Yes	1.21 (1.07–1.38)	0.004	1.18 (1.03–1.36)	0.020	1.13 (0.98–1.30)	0.093
Adulthood social isolation						
No	Ref	—	Ref	—	Ref	—
Yes	1.26 (1.06–1.51)	0.011	1.17 (0.97–1.41)	0.097	1.13 (0.94–1.37)	0.194
Social isolation trajectories						
No isolation	Ref	—	Ref	—	Ref	—
Childhood only	1.17 (1.01–1.34)	0.036	1.13 (0.97–1.32)	0.115	1.08 (0.93–1.26)	0.331
Adulthood only	1.14 (0.90–1.45)	0.276	1.04 (0.81–1.34)	0.742	1.01 (0.78–1.29)	0.965
Persistent isolation	1.64 (1.25–2.13)	<0.001	1.53 (1.16–2.01)	0.003	1.42 (1.08–1.88)	0.013

*Note*: Model 1: Unadjusted. Model 2: Adjust for age, sex, education, residence, BMI, smoking, drinking, hypertension, diabetes, dyslipidemia,.Childhood economic status, childhood health status. Model 3: Adjust for age, sex, education, residence, BMI, smoking, drinking, hypertension, diabetes, dyslipidemia,. Childhood economic status, childhood health status, depressive symptoms.

Additional analyses examined associations between social isolation at different life stages and social isolation trajectories with incident heart disease (Table S3) and incident stroke (Table S4). For incident heart disease, persistent isolation was associated with elevated odds in the unadjusted model (OR 1.40, 95% CI 1.03–1.90); however, this association was no longer statistically significant in the fully adjusted Model 2 (*p* = 0.116). In contrast, social isolation trajectories demonstrated a stronger and more consistent association with incident stroke. In Model 2, persistent isolation remained significantly associated with an increased odds of incident stroke (OR 1.79, 95% CI 1.19–2.71). Notably, childhood social isolation was also independently associated with higher odds of incident stroke (OR 1.44, 95% CI 1.16–1.79).

### 3.3. Mediation Analysis

Given that the inclusion of depressive symptoms led to an attenuation of the main effect estimate (Model 2 OR 1.53 vs Model 3 OR 1.42), mediation analyses were conducted (Figure 3). The results indicated that depressive symptoms partially mediated the association between persistent social isolation and incident CVD, accounting for 16.28% of the total effect (*p* < 0.001). A comparable mediating pattern was observed for incident stroke, with depressive symptoms explaining 14.70% of the overall association (*p* < 0.001).

Figure 3Mediation analysis of depressive symptoms linking persistent social isolation to incident CVD (A) and stroke (B).

**Figure:**
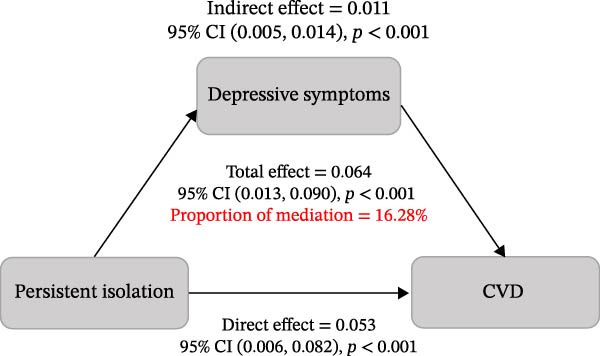
(A)

**Figure:**
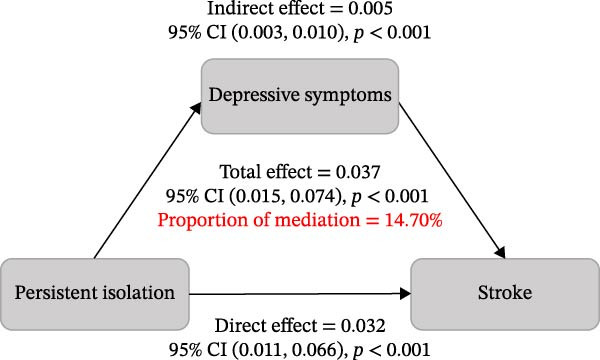
(B)

### 3.4. Subgroup and Sensitivity Analyses

Subgroup analyses demonstrated substantial consistency in the association between social isolation trajectories and incident CVD across strata defined by sex, residence, and depressive symptoms, with no statistically significant interactions detected (all *p*‐values for interaction >0.05; Figure S3). These findings suggest that the detrimental impact of persistent social isolation is broadly evident across population subgroups with differing sociodemographic and psychological profiles.

In Cox proportional hazards models incorporating follow‐up time, persistent isolation remained significantly associated with an increased risk of incident CVD (HR 1.42, 95% CI 1.12–1.80), consistent in direction and magnitude with the primary logistic regression results (Table S5). Similarly, complete‐case analyses excluding participants with missing covariate data yielded a comparable odds estimate for persistent isolation (OR 1.54, 95% CI 1.15–2.07), closely aligning with findings from the multiple imputation–based main analysis (Table S6). Finally, E‐value analyses indicated that the observed association (OR 1.53) corresponded to an E‐value of 2.43, with an E‐value of 1.59 for the lower bound of the CI. This implies that an unmeasured confounder would need to be associated with both persistent social isolation and incident CVD by a risk ratio of at least 2.43 to fully account for the observed association, thereby providing additional support for the robustness of the primary findings.

## 4. Discussion

Using nationally representative cohort data, this study provides the first comprehensive evaluation of associations between social isolation trajectories from childhood through adulthood and the risk of incident CVD among Chinese adults aged 45 years and older, while also examining the potential mediating role of depressive symptoms. Four distinct life‐course patterns of social isolation were identified, with individuals experiencing persistent isolation facing the highest risk of CVD onset. Importantly, depressive symptoms were found to partially mediate this relationship. Collectively, these findings advance understanding of the cumulative, life‐course health consequences of social isolation, offer empirical support for earlier cardiovascular risk stratification, and suggest that interventions targeting depressive symptoms may represent a critical leverage point for potentially modifying the pathways linking social isolation to CVD.

Accumulating evidence suggests that the dynamic evolution of social isolation may be more strongly associated with health outcomes than static assessments at a single time point. For example, Zhang et al. [25], drawing on two prospective cohorts, CHARLS and SHARE, reported that persistent social isolation was significantly associated with an increased risk of frailty among older adults (CHARLS: HR 1.2, 95% CI 1.1–1.4; SHARE: HR 1.4, 95% CI 1.2–1.6). Using a life‐course approach, Lay‐Yee et al. [26] constructed social isolation trajectories from childhood through midlife and demonstrated a robust association between persistent isolation and a markedly elevated risk of adult depression (OR 4.20, 95% CI 1.84–9.61). Similarly, Guo et al. conducted a longitudinal analysis using CHARLS data and found that middle‐aged and older adults exposed to sustained social isolation had a 45% higher risk of developing CVD (HR 1.45, 95% CI 1.13–1.85) [27]. Building on this growing literature, the present study extends the dynamic perspective by integrating retrospective childhood information to construct social isolation trajectories spanning the entire life course. In contrast to the work of Guo et al., which primarily focused on isolation during adulthood, our findings underscore childhood as a critical risk origin. Specifically, although isolation confined to either childhood or adulthood alone did not confer a statistically significant excess risk after full adjustment, persistent isolation across both life stages was robustly associated with an increased risk of incident CVD. These results suggest that cardiovascular vulnerability is not solely a consequence of social deprivation in later life but rather reflects the cumulative interplay of adverse social exposures across the lifespan. From a life‐course epidemiology perspective, this pattern aligns strongly with the cumulative exposure model. The lack of significant associations for transient, single‐stage isolation suggests that temporary psychosocial deprivation may not accumulate a sufficient “dose” to induce irreversible cardiovascular damage. Indeed, subsequent improvements in social environments may provide a critical buffering effect that mitigates early physiological perturbations. Conversely, persistent isolation generates sustained physiological wear–and–tear, or allostatic load, across critical developmental and aging windows, eventually breaching the threshold for clinical CVD.

Interestingly, analyses focusing on incident stroke yielded a pattern distinct from that observed for overall CVD. Both childhood social isolation and the childhood‐only isolation trajectory were significantly associated with an elevated risk of stroke. This finding lends strong support to the notion that experiences of social exclusion during critical developmental periods may induce lasting biological alterations, potentially through remodeling of neuroendocrine pathways and epigenetic modifications, thereby leaving durable imprints on physiological systems [28, 29]. Such early‐life biological perturbations may exert a cumulative and preferentially deleterious effect on the cerebrovascular system, increasing vulnerability to stroke in later life. Nevertheless, it is noteworthy that persistent isolation conferred the highest risk for both stroke and overall CVD. This pattern suggests that although childhood isolation may establish a foundation of physiological susceptibility, it does not constitute an irreversible life‐course trajectory [30, 31]. Improvements in social environments during adulthood may provide a crucial buffering effect, partially mitigating the cardiovascular and cerebrovascular consequences of early‐life isolation. Collectively, these observations underscore the importance of addressing adverse social isolation trajectories and fostering social connectedness across the entire life span.

The mediation analysis further revealed that the cumulative burden of social isolation is associated with an increased CVD risk, in part through detrimental effects on mental health, specifically by inducing depressive symptoms. In the present study, depressive symptoms accounted for 16.28% of the total effect for incident CVD and 14.70% for incident stroke. These findings are highly biologically plausible. Depressive symptoms are not only associated with adverse health behaviors but are also directly linked to autonomic nervous system dysregulation, platelet activation, and elevated levels of pro‐inflammatory cytokines [32–34]. Thus, depressive symptoms may represent more than a psychological consequence of social isolation; they may function as a key physiological bridge linking adverse social environments to cardiovascular pathology [35]. However, the modest mediation proportion indicates that depressive symptoms only partially explain this complex association. The substantial remaining effect is likely driven by other unmeasured mechanisms. For instance, persistent social isolation may lead to the adoption of unhealthy lifestyle behaviors, such as physical inactivity and poor diet.

These findings suggest that assessment of social connections at a single time point may be insufficient to fully capture cardiovascular risk. Clinicians should consider incorporating questions about life‐course social support and isolation histories into routine medical assessments. Moreover, social isolation can be evaluated using simple, low‐cost questionnaires, making it a feasible and practical tool for identifying high‐risk individuals in primary care settings. Finally, given that depressive symptoms account for a modest portion of the risk, clinical interventions must be multidimensional. While routine screening for and treatment of depressive symptoms remain crucial for individuals exposed to long‐term social isolation, secondary prevention strategies should simultaneously incorporate direct efforts to promote social engagement and rigorously manage metabolic risks, thereby comprehensively interrupting the progression toward CVD.

Several limitations of this study should be acknowledged. First, the observational design precludes definitive causal inference regarding the relationship between social isolation and incident CVD. Furthermore, the temporal relationship between adulthood social isolation and depressive symptoms must be interpreted with caution. Because both variables were assessed concurrently at the 2015 baseline, we cannot entirely rule out a bidirectional relationship. Future longitudinal studies with repeated measures across multiple time points are required to rigorously confirm the exact causal sequence linking social isolation, depressive symptoms, and incident CVD. Second, childhood social isolation was assessed retrospectively via questionnaire, which may be subject to recall bias, a common challenge in life‐course research [36, 37]. Third, CVD outcomes were based on self‐reported physician diagnoses. While prior studies have demonstrated the reliability of such measures within CHARLS, some degree of misclassification remains possible [38, 39]. Finally, although the absolute number of incident events in the persistent isolation group was statistically sufficient for robust multivariable modeling, this specific trajectory represented a relatively small proportion of the total cohort. Therefore, our findings warrant further replication in larger, diverse populations to ensure broader generalizability.

## 5. Conclusion

In summary, this study provides robust evidence that persistent social isolation spanning childhood and adulthood is significantly associated with an increased risk of incident CVD among middle‐aged and older adults, with this relationship partially mediated by depressive symptoms. These findings underscore the importance of adopting a life‐course perspective in addressing psychosocial health and suggest that interventions aimed at disrupting social isolation trajectories and timely management of depressive symptoms may hold substantial potential for reducing CVD risk.

## Author Contributions

Shuaiqing Chen and Qiuxia Zheng participated in data collection and drafting and editing of the paper. Peiling Jiang participated in data analysis and revision of the paper. All authors saw and approved the final version, and no other person made a substantial contribution to the paper.

## Funding

This work was supported by the Zhejiang Province Traditional Chinese Medicine Science and Technology Plan Project (Project 2025ZL611), and Zhejiang Province Traditional Chinese Medicine Science and Technology Plan Project (Project 2025ZL616).

## Disclosure

The funding body did not participate in the design of the study, data collection, analysis, and interpretation of data or in writing the manuscript.

## Ethics Statement

This study was conducted in accordance with the Declaration of Helsinki. CHARLS was approved by the Institutional Review Board of Peking University (approval number: IRB00001052‐11015 for the household survey and IRB00001052‐11014 for blood samples), and all participants provided written informed consent.

## Consent

The authors have nothing to report.

## Conflicts of Interest

The authors declare no conflicts of interest.

## Supporting Information

Additional supporting information can be found online in the Supporting Information section.

## Supporting information


**Supporting Information** Table S1: Baseline characteristics of excluded and included participants. Table S2: Variance inflation factors for covariates. Table S3: Association of social isolation trajectories with incident heart disease. Table S4: Association of social isolation trajectories with incident stroke. Table S5: Association of social isolation trajectories with incident CVD using Cox proportional hazards models. Table S6: Association of social isolation trajectories with incident CVD after excluding participants with missing covariates. Figure S1: Proportion of missing values for baseline covariates. Figure S2: Sankey diagram illustrating the life‐course social isolation trajectories from childhood to adulthood. Figure S3: Subgroup analyses of the association between social isolation trajectories and incident CVD.

## Data Availability

The data supporting the findings of this study are available on the CHARLS website (http://charls.pku.edu.cn/).
