# Supplementary material for: Associations of Life‐Course Social Isolation Trajectories and Depressive Symptoms With the Risk of Incident Cardiovascular Disease: A Prospective Cohort Study
Source: Depress Anxiety. 2026 Apr 8;2026:2184277. doi: 10.1155/da/2184277 (PMC13059090; doi:10.1155/da/2184277)

**Table S1. Baseline characteristics of excluded and included participants**

| **Variables** | **Total** | **Excluded Participants**  **(n = 14239)** | **Included** **Participants**  **(n = 6858)** | ***P*** |
| --- | --- | --- | --- | --- |
|  | **(n = 21097)** |  |  |  |
| **Age, years** | 59.09 ± 10.75 | 59.04 ± 11.70 | 59.20 ± 8.46 | 0.260 |
| **BMI, kg/m²** | 24.55 ± 23.84 | 25.06 ± 31.21 | 23.85 ± 3.83 | <0.001 |
| **Sex, n (%)** |  |  |  | <0.001 |
| Female | 10062 (47.71) | 6261 (43.99) | 3801 (55.42) |  |
| Male | 11030 (52.29) | 7973 (56.01) | 3057 (44.58) |  |
| **Education, n (%)** |  |  |  | <0.001 |
| Below elementary school | 8587 (40.74) | 6548 (46.05) | 2039 (29.73) |  |
| Elementary school | 6224 (29.53) | 4228 (29.73) | 1996 (29.10) |  |
| Above elementary school | 6266 (29.73) | 3443 (24.21) | 2823 (41.16) |  |
| **Residence, n (%)** |  |  |  | <0.001 |
| Urban | 4155 (22.63) | 2908 (24.43) | 1247 (19.31) |  |
| Rural | 14207 (77.37) | 8996 (75.57) | 5211 (80.69) |  |
| **Smoking, n (%)** |  |  |  | <0.001 |
| No | 15056 (71.97) | 10429 (74.15) | 4627 (67.49) |  |
| Yes | 5864 (28.03) | 3635 (25.85) | 2229 (32.51) |  |
| **Drinking, n (%)** |  |  |  | <0.001 |
| No | 13507 (64.59) | 9408 (66.91) | 4099 (59.82) |  |
| Yes | 7406 (35.41) | 4653 (33.09) | 2753 (40.18) |  |
| **Hypertension, n (%)** |  |  |  | <0.001 |
| No | 11248 (66.13) | 6286 (61.27) | 4962 (73.52) |  |
| Yes | 5760 (33.87) | 3973 (38.73) | 1787 (26.48) |  |
| **Diabetes, n (%)** |  |  |  | <0.001 |
| No | 15207 (90.10) | 8982 (88.63) | 6225 (92.32) |  |
| Yes | 1670 (9.90) | 1152 (11.37) | 518 (7.68) |  |
| **Dyslipidemia, n (%)** |  |  |  | <0.001 |
| No | 13467 (81.30) | 7830 (78.87) | 5637 (84.93) |  |
| Yes | 3098 (18.70) | 2098 (21.13) | 1000 (15.07) |  |
| **Depressive symptoms, n (%)** |  |  |  | <0.001 |
| No | 13255 (67.59) | 8413 (65.93) | 4842 (70.68) |  |
| Yes | 6356 (32.41) | 4347 (34.07) | 2009 (29.32) |  |
| **Childhood economic status, n (%)** |  |  |  | <0.001 |
| Good | 1774 (9.56) | 1150 (9.82) | 624 (9.11) |  |
| Fair | 9462 (51.00) | 5834 (49.84) | 3628 (52.98) |  |
| Poor | 7317 (39.44) | 4721 (40.33) | 2596 (37.91) |  |
| **Childhood health status, n (%)** |  |  |  | <0.001 |
| Good | 6550 (35.33) | 4031 (34.47) | 2519 (36.81) |  |
| Fair | 9567 (51.61) | 6051 (51.75) | 3516 (51.37) |  |
| Poor | 2420 (13.05) | 1611 (13.78) | 809 (11.82) |  |

Data are presented as mean ± standard deviation (SD) or number (%), as appropriate

The *P*-value is based on analysis of variance (ANOVA), chi-square test, or Fisher's exact test

Abbreviations: BMI, body mass index

**Table S2. Variance inflation factors for covariates**

| **Variables** | **VIF** |
| --- | --- |
| **Social isolation trajectories** | 1.016 |
| **Age** | 1.098 |
| **Sex** | 1.328 |
| **Education** | 1.063 |
| **Residence** | 1.056 |
| **Smoking** | 1.21 |
| **Drinking** | 1.124 |
| **Hypertension** | 1.062 |
| **Diabetes** | 1.041 |
| **Depressive symptoms** | 1.04 |
| **Dyslipidemia** | 1.058 |
| **Childhood economic status** | 1.02 |
| **Childhood health status** | 1.014 |
| **BMI** | 1.073 |

Abbreviations: BMI, body mass index

**Table S3. Association of social isolation trajectories with incident heart disease**

| **Variables** | **Model1** | |  | **Model2** | |  | **Model3** | |
| --- | --- | --- | --- | --- | --- | --- | --- | --- |
|  | **OR (95%CI)** | ***P*** |  | **OR (95%CI)** | ***P*** |  | **OR (95%CI)** | ***P*** |
| **Childhood social isolation** |  |  |  |  |  |  |  |  |
| No | Ref |  |  | Ref |  |  | Ref |  |
| Yes | 1.07 (0.92-1.24) | 0.370 |  | 1.04 (0.89-1.22) | 0.650 |  | 1.00 (0.85-1.17) | 0.993 |
| **Adulthood social isolation** |  |  |  |  |  |  |  |  |
| No | Ref |  |  | Ref |  |  | Ref |  |
| Yes | 1.28 (1.05-1.57) | 0.015 |  | 1.18 (0.96-1.45) | 0.114 |  | 1.15 (0.93-1.42) | 0.189 |
| **Social isolation trajectories** |  |  |  |  |  |  |  |  |
| No isolation | Ref |  |  | Ref |  |  | Ref |  |
| Childhood only | 1.04 (0.89-1.23) | 0.603 |  | 1.01 (0.85-1.20) | 0.897 |  | 0.97 (0.82-1.16) | 0.768 |
| Adulthood only | 1.23 (0.95-1.60) | 0.110 |  | 1.12 (0.86-1.47) | 0.392 |  | 1.09 (0.83-1.43) | 0.523 |
| Persistent isolation | 1.40 (1.03-1.90) | 0.029 |  | 1.29 (0.94-1.76) | 0.116 |  | 1.22 (0.89-1.67) | 0.228 |

Model1: Unadjusted

Model2: Adjust for Age, Sex, Education, Residence, BMI, Smoking, Drinking, Hypertension, Diabetes, Dyslipidemia,

Childhood economic status, Childhood health status

Model3: Adjust for Age, Sex, Education, Residence, BMI, Smoking, Drinking, Hypertension, Diabetes, Dyslipidemia,

Childhood economic status, Childhood health status, Depressive symptoms

**Table S4. Association of social isolation trajectories with incident stroke**

| **Variables** | **Model1** | |  | **Model2** | |  | **Model3** | |
| --- | --- | --- | --- | --- | --- | --- | --- | --- |
|  | **OR (95%CI)** | ***P*** |  | **OR (95%CI)** | ***P*** |  | **OR (95%CI)** | ***P*** |
| **Childhood social isolation** |  |  |  |  |  |  |  |  |
| No | Ref |  |  | Ref |  |  | Ref |  |
| Yes | 1.48 (1.21-1.81) | <0.001 |  | 1.44 (1.16-1.79) | <0.001 |  | 1.35 (1.09-1.68) | 0.007 |
| **Adulthood social isolation** |  |  |  |  |  |  |  |  |
| No | Ref |  |  | Ref |  |  | Ref |  |
| Yes | 1.18 (0.88-1.57) | 0.260 |  | 1.11 (0.82-1.49) | 0.498 |  | 1.06 (0.79-1.43) | 0.711 |
| **Social isolation trajectories** |  |  |  |  |  |  |  |  |
| No isolation | Ref |  |  | Ref |  |  | Ref |  |
| Childhood only | 1.41 (1.13-1.76) | 0.002 |  | 1.36 (1.08-1.72) | 0.010 |  | 1.28 (1.01-1.62) | 0.044 |
| Adulthood only | 0.99 (0.66-1.50) | 0.972 |  | 0.91 (0.60-1.39) | 0.677 |  | 0.87 (0.57-1.32) | 0.510 |
| Persistent isolation | 1.86 (1.25-2.75) | 0.002 |  | 1.79 (1.19-2.71) | 0.005 |  | 1.64 (1.08-2.48) | 0.020 |

Model1: Unadjusted

Model2: Adjust for Age, Sex, Education, Residence, BMI, Smoking, Drinking, Hypertension, Diabetes, Dyslipidemia,

Childhood economic status, Childhood health status

Model3: Adjust for Age, Sex, Education, Residence, BMI, Smoking, Drinking, Hypertension, Diabetes, Dyslipidemia,

Childhood economic status, Childhood health status, Depressive symptoms

**Table S5. Association of social isolation trajectories with incident CVD using Cox proportional hazards models**

| **Variables** | **Model1** | |  | **Model2** | |  | **Model3** | | |
| --- | --- | --- | --- | --- | --- | --- | --- | --- | --- |
|  | **HR (95%CI)** | ***P*** |  | **HR (95%CI)** | ***P*** |  | **HR (95%CI)** | | ***P*** |
| **Childhood social isolation** |  |  |  |  |  |  |  | |  |
| No | Ref |  |  | Ref |  |  | Ref | |  |
| Yes | 1.18 (1.05-1.33) | 0.005 |  | 1.14 (1.01-1.29) | 0.030 |  | | 1.10 (0.97-1.24) | 0.127 |
| **Adulthood social isolation** |  |  |  |  |  |  | |  |  |
| No | Ref |  |  | Ref |  |  | | Ref |  |
| Yes | 1.23 (1.04-1.44) | 0.013 |  | 1.14 (0.97-1.35) | 0.104 |  | | 1.11 (0.95-1.31) | 0.202 |
| **Social isolation trajectories** |  |  |  |  |  |  | |  |  |
| No isolation | Ref |  |  | Ref |  |  | | Ref |  |
| Childhood only | 1.14 (1.01-1.30) | 0.044 |  | 1.10 (0.96-1.26) | 0.153 |  | | 1.06 (0.93-1.21) | 0.407 |
| Adulthood only | 1.13 (0.91-1.40) | 0.281 |  | 1.04 (0.83-1.29) | 0.745 |  | | 1.00 (0.81-1.25) | 0.971 |
| Persistent isolation | 1.52 (1.21-1.92) | <0.001 |  | 1.42 (1.12-1.80) | 0.004 |  | | 1.34 (1.06-1.70) | 0.015 |

Model1: Unadjusted

Model2: Adjust for Age, Sex, Education, Residence, BMI, Smoking, Drinking, Hypertension, Diabetes, Dyslipidemia,

Childhood economic status, Childhood health status

Model3: Adjust for Age, Sex, Education, Residence, BMI, Smoking, Drinking, Hypertension, Diabetes, Dyslipidemia,

Childhood economic status, Childhood health status, Depressive symptoms

**Table S6. Association of social isolation trajectories with incident CVD after excluding participants with missing covariates**

| **Variables** | **Model1** | |  | **Model2** | |  | **Model3** | | |
| --- | --- | --- | --- | --- | --- | --- | --- | --- | --- |
|  | **OR (95%CI)** | ***P*** |  | **OR (95%CI)** | ***P*** |  | **OR (95%CI)** | | ***P*** |
| **Childhood social isolation** |  |  |  |  |  |  |  | |  |
| No | Ref |  |  | Ref |  |  | Ref | |  |
| Yes | 1.20 (1.04-1.38) | 0.012 |  | 1.15 (0.99-1.33) | 0.071 |  | | 1.10 (0.94-1.28) | 0.233 |
| **Adulthood social isolation** |  |  |  |  |  |  | |  |  |
| No | Ref |  |  | Ref |  |  | | Ref |  |
| Yes | 1.31 (1.09-1.59) | 0.005 |  | 1.21 (0.99-1.48) | 0.061 |  | | 1.18 (0.96-1.44) | 0.112 |
| **Social isolation trajectories** |  |  |  |  |  |  | |  |  |
| No isolation | Ref |  |  | Ref |  |  | | Ref |  |
| Childhood only | 1.14 (0.98-1.33) | 0.094 |  | 1.09 (0.93-1.29) | 0.290 |  | | 1.04 (0.88-1.23) | 0.614 |
| Adulthood only | 1.18 (0.92-1.52) | 0.197 |  | 1.07 (0.82-1.39) | 0.617 |  | | 1.04 (0.80-1.35) | 0.767 |
| Persistent isolation | 1.68 (1.27-2.22) | <0.001 |  | 1.54 (1.15-2.07) | 0.004 |  | | 1.44 (1.07-1.94) | 0.016 |

Model1: Unadjusted

Model2: Adjust for Age, Sex, Education, Residence, BMI, Smoking, Drinking, Hypertension, Diabetes, Dyslipidemia,

Childhood economic status, Childhood health status

Model3: Adjust for Age, Sex, Education, Residence, BMI, Smoking, Drinking, Hypertension, Diabetes, Dyslipidemia,

Childhood economic status, Childhood health status, Depressive symptoms

**Figure S1. Proportion of missing values for baseline covariates**


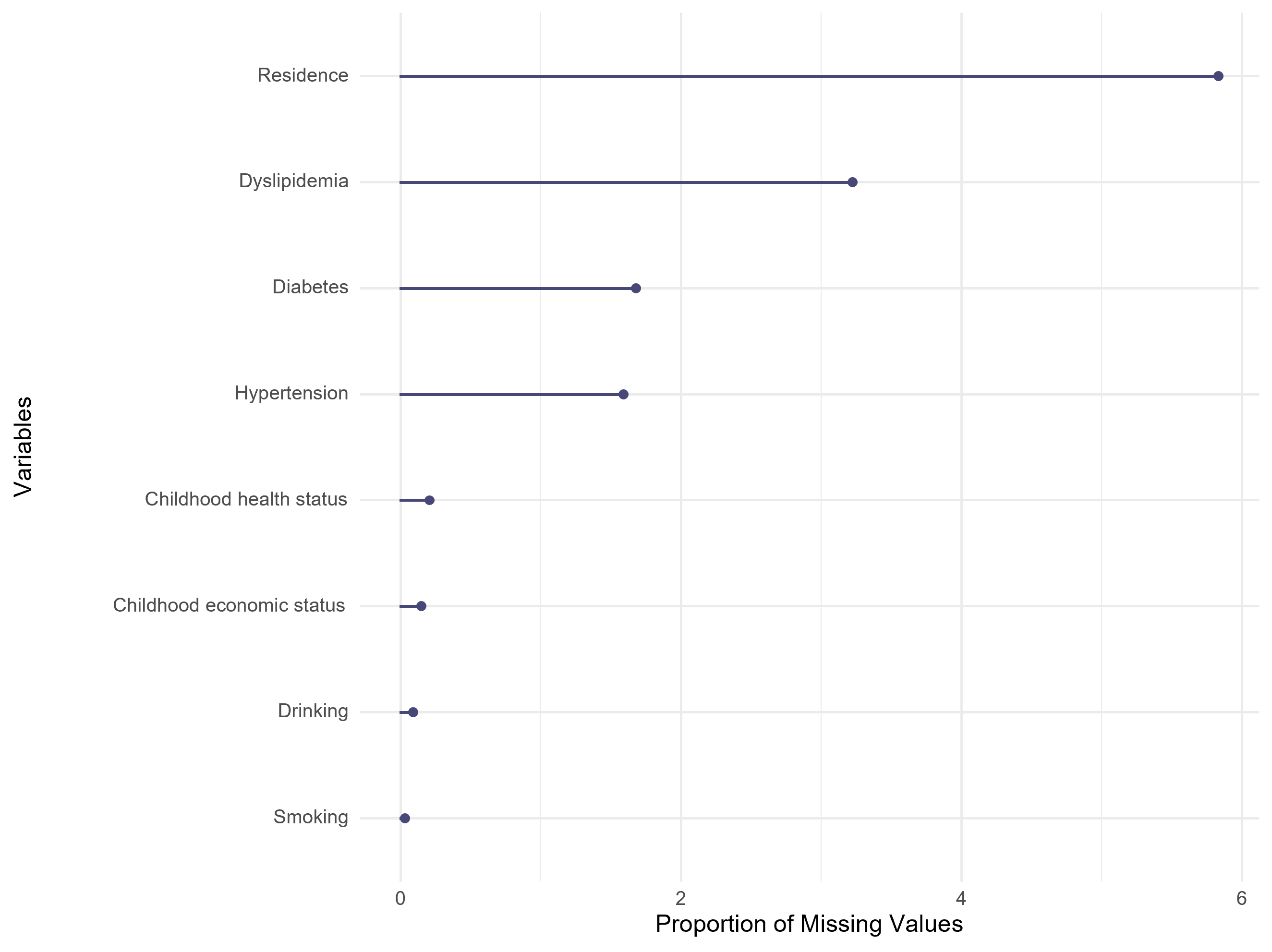


**Figure S2. Sankey diagram illustrating the life-course social isolation trajectories from childhood to adulthood**


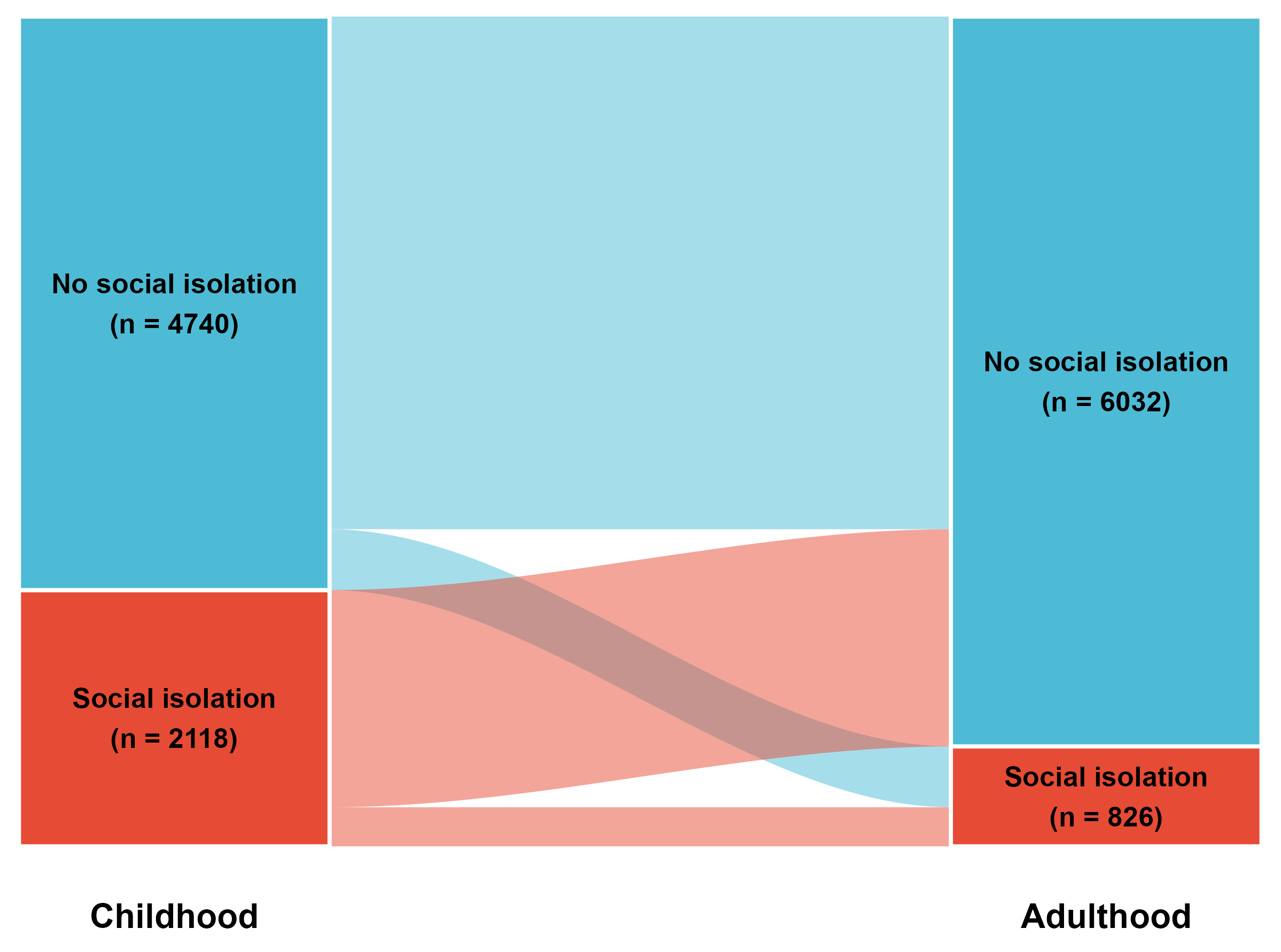


**Figure S3. Subgroup analyses of the association between social isolation trajectories and incident CVD**


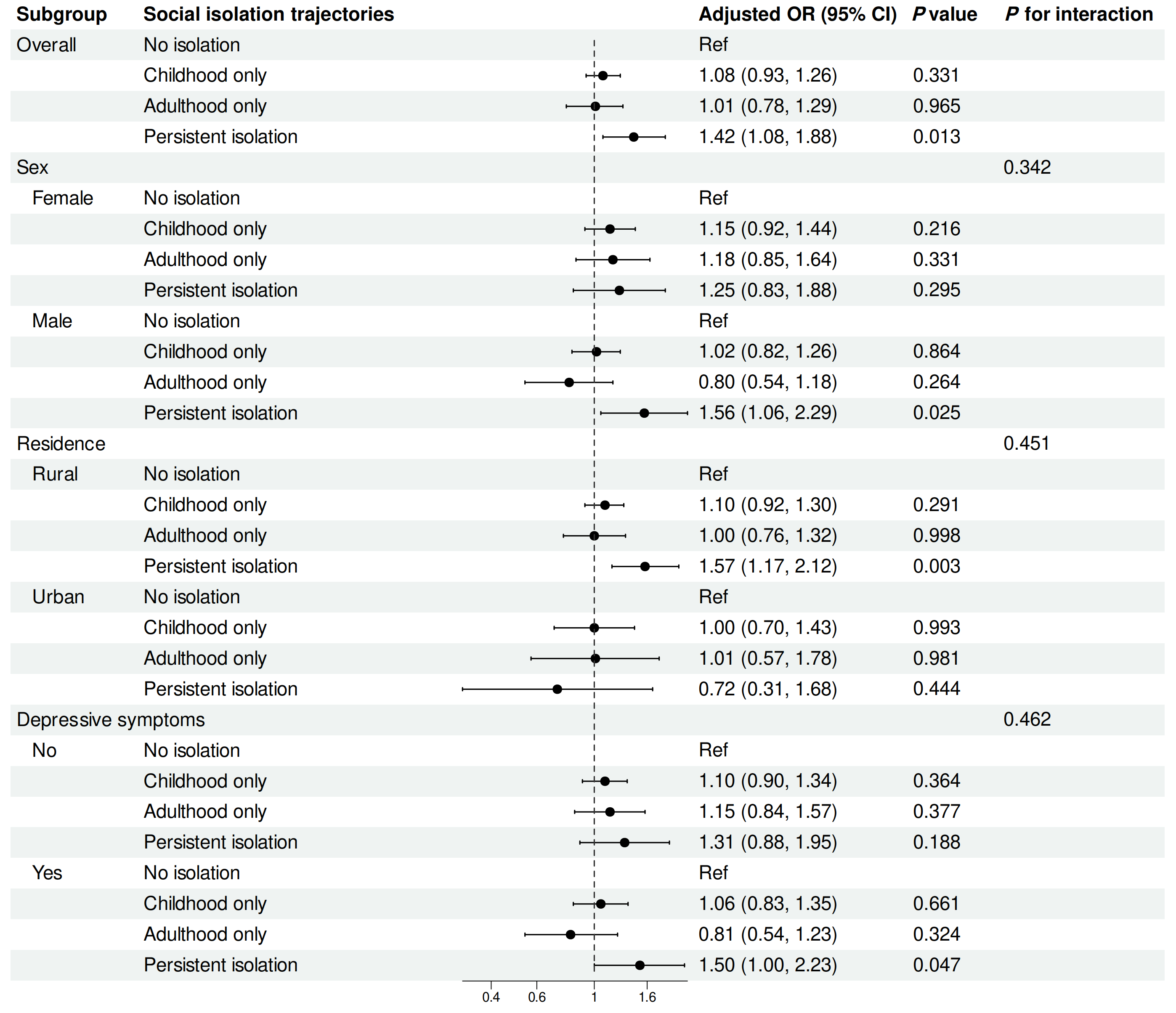

Supplement: Supplementary file 1 — Supporting Information Table S1: Baseline characteristics of excluded and included participants. Table S2: Variance inflation factors for covariates. Table S3: Association of social isolation trajectories with incident heart disease. Table S4: Association of social isolation trajectories with incident stroke. Table S5: Association of social isolation trajectories with incident CVD using Cox proportional hazards models. Table S6: Association of social isolation trajectories with incident CVD after excluding participants with missing covariates. Figure S1: Proportion of missing values for baseline covariates. Figure S2: Sankey diagram illustrating the life‐course social isolation trajectories from childhood to adulthood. Figure S3: Subgroup analyses of the association between social isolation trajectories and incident CVD. [file DA-2026-2184277-s001.docx]
